# Privileged substructures for anti-sickling activity *via* cheminformatic analysis†

**DOI:** 10.1039/c7ra12079f

**Published:** 2018-02-07

**Authors:** Chuleeporn Phanus-umporn, Watshara Shoombuatong, Veda Prachayasittikul, Nuttapat Anuwongcharoen, Chanin Nantasenamat

**Affiliations:** Center of Data Mining and Biomedical Informatics, Faculty of Medical Technology, Mahidol University Bangkok 10700 Thailand chanin.nan@mahidol.edu

## Abstract

Sickle cell disease (SCD), an autosomal recessive genetic disorder, has been recognized by the World Health Organization (WHO) as a major public health problem as it affects 300 000 individuals worldwide. Complications arising from SCD include anemia, microvascular occlusion, severe pain, stokes, renal dysfunction and infections. A lucrative therapeutic strategy is to employ anti-sickling agents that can disrupt the formation of the HbS polymer. This study therefore employed cheminformatic approaches, encompassing classification structure–activity relationship (CSAR) modeling, to deduce the privileged substructures giving rise to the anti-sickling activity of an investigated set of 115 compounds, followed by substructure analysis. Briefly, the compiled compounds were described by fingerprint descriptors and used in the construction of CSAR models *via* several machine learning algorithms. The modelability of the data set, as exemplified by the MODI index, was determined to be in the range of 0.70–0.84. The predictive performance was deduced by the accuracy, sensitivity, specificity and Matthews correlation coefficient, which was found to be statistically robust, whereby the former three parameters afforded values in excess of 0.7 while the latter statistical parameter provided a value greater than 0.5. An analysis of the top 20 important substructure descriptors for anti-sickling activity revealed that 10 important features were significant in the differentiation of actives from inactives, as illustrated by aromaticity/conjugation (*e.g.* SubFPC287, SubFPC171 and SubFPC5), carbonyl groups (*e.g.* SubFPC137, SubFPC139, SubFPC49 and SubFPC135) and miscellaneous groups (*e.g.* SubFPC303, SubFPC302 and SubFPC275). Furthermore, an analysis of the structure–activity relationship revealed that the length of alkyl chains, choice of functional moiety and position of substitution on the benzene ring may affect the anti-sickling activity of these compounds. Thus, this knowledge is anticipated to be useful for guiding the design of robust compounds against the gelling activity of HbS, as preliminarily demonstrated in the data-driven compound design presented herein.

## Abstract 2

Cheminformatic approaches (classification structure–activity relationship models based on 12 fingerprint classes) were employed for deducing privileged substructures giving rise to the anti-sickling activity of an investigated set of 115 compounds.

## Graphical abstract

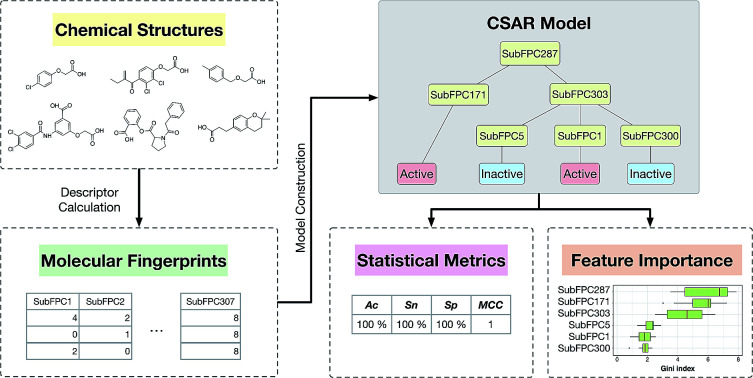

## 1 Introduction

Human hemoglobin (Hb) is an iron-containing protein that is found abundantly within red blood cells (RBCs). Hb is formed by a symmetric polypeptide chain dimer pairing in which the α-like and β-like chains form a tetrameric structural and functional unit. Their main function is to transport O_2_ from the lungs to all body tissues, as well as to transport CO_2_ out of the tissues and back to the lungs. Furthermore, Hb is also capable of interacting with other gases, such as carbon monoxide (CO) and nitric oxide (NO), and these interactions govern important biological roles.^1^ Adult hemoglobin (HbA) is the most common form of hemoglobin in adults and is composed of two α-chains and two β-chains, constituting 141 and 146 amino acids, respectively.^2^ Mutations of the genes result in the structural alteration and perturbation of the globin chain that eventually culminates in Hb-associated diseases as seen in HbA, hemoglobin S (HbS), hemoglobin C (HbC) and hemoglobin E (HbE), as well as thalassemia (*i.e.* decreased globin chain production).^3^

Sickle cell disease (SCD) is a global health problem in several parts of the world (*e.g.* sub-Saharan Africa, the Mediterranean basin, the Middle East, India and the United States) that has been estimated to annually affect approximately 300 000 infants (WHO), and this number has been forecasted to rise to 400 000 by 2050.^4^ The hallmark of SCD involves the polymerization of deoxygenated HbS that consequently leads to the sickling process that alters the shape of RBCs.^5^ Mechanistically, HbS arises from the A → T point mutation that leads to the substitution of hydrophilic glutamic acid with hydrophobic valine at the sixth position (Glu6Val) of the β-globin chain.^6^ The resulting Val6 on the β_2_-globin chain then interacts hydrophobically with Phe85 and Leu88 from a neighboring Hb molecule. At low oxygen tension, HbS is polymerized inside RBCs, leading to gel or fiber formation and thereby causing a drastic decrease in red cell deformability. Consequently, this leads to several complications such as anemia, microvascular occlusion, severe pain, strokes, renal dysfunction and infections.

**Fig. 1:**
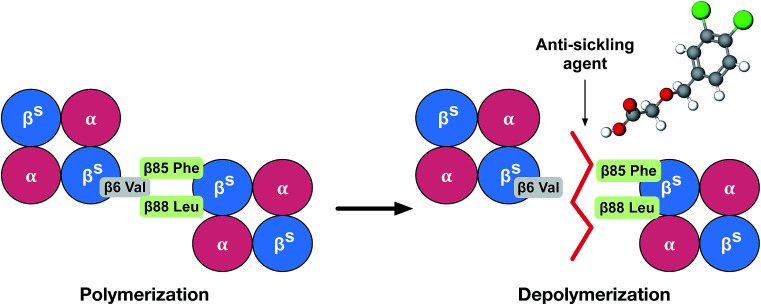
Cartoon illustration of the mechanism of action of an anti-sickling agent in the disruption of the HbS polymer.

Currently, the clinical management of SCD is blood transfusion, although long-term transfusion therapy may cause an iron overload in patients, leading to potential side effects such as organ damage and infections. Even though iron chelation therapy has greatly improved blood transfusion, it only offers a temporary solution to the problem.^7^ Allogeneic hematopoietic stem cell transplantation (HSCT) is a gene transfer therapy aimed at the underlying molecular causes of SCD. However, most successful transplantations require the use of stem cells from matched sibling donors, thereby making this a challenging therapeutic approach for some patients. HSCT may therefore not be applicable for many current patients.^8^ Gene therapy is one of the most promising approaches as it does not try to fix the symptoms, but rather the problem of the disease.^9^ However, this approach is available to only a very small percentage of patients due to its extremely high costs and requirements for highly specialized centers. Moreover, several anti-sickling agents have been investigated and confirmed to possess ameliorative properties. Hydroxyurea has been shown to decrease the number and severity of sickled cells by significantly increasing fetal hemoglobin (HbF) production in patients with SCD. It was therefore approved for use by the FDA in 1998. Nevertheless, the side effects of this drug include neutropenia, bone marrow suppression, elevation of hepatic enzymes, anorexia, nausea, vomiting and infertility.^10,11^ Recently, on July 7, 2017, the U.S. FDA approved l-glutamine oral powder (Endari) as the first new drug in 20 years for SCD, which acts by reducing acute complications in adults and children of 5 years and older. Although its mechanism of action is not fully understood, the drug is found to be involved in the oxidative stress phenomena of SCD. It has been shown to improve the nicotinamide adenine dinucleotide (NAD) redox potential of RBCs by increasing the availability of reduced glutathione. However, several common adverse reactions were found in >10% of incidences, such as nausea, headaches, abdominal pain, coughs, pain in the extremities, back pain and chest pain.^12^ In fact, it should be noted that several side effects with no specific therapy occur for SCD patients. Therefore, the pathophysiological hallmark of SCD presents an interesting subject. The idea for the treatment of SCD was to bind small molecules near the mutation site in such a fashion that it would prevent the insertion of the β-globin chain of Hb containing the Val mutation (the donor site) into the hydrophobic pocket of a second Hb molecule (the acceptor site), thereby inhibiting deoxy-HbS polymerization (Fig. 1). The rationale for our study was to follow the treatment of SCD based on pathophysiology, to inhibit deoxy-HbS polymerization using computational methods.

Classification structure–activity relationship (CSAR) modeling represents an important approach for elucidating the origin of biological activity for a set of compounds of interest as a function of their molecular descriptors. The obtained CSAR model can help to reveal the privileged substructures that are essential for the biological activity of potent compounds which can subsequently be used as therapeutic agents. Privileged substructures are a concept introduced by Evans *et al.*^13^ in their analysis of cholecystokinin antagonists based on benzodiazepines, in which they discovered that there exist conserved substructures that were not found in compounds of different activity. Therefore, we applied CSAR, together with scaffold and substructure analysis, to rationalize the underlying physicochemical features defining anti-sickling activity in several series of compounds reported by Abraham and colleagues.^14–20^ In this study, we examined the utility of several sets of substructure fingerprint descriptors in modeling anti-sickling activity. Important physicochemical features were then decoded from such predictive CSAR models to discern the privileged substructures influencing the anti-sickling activity.

## 2 Materials and methods

**Fig. 2:**
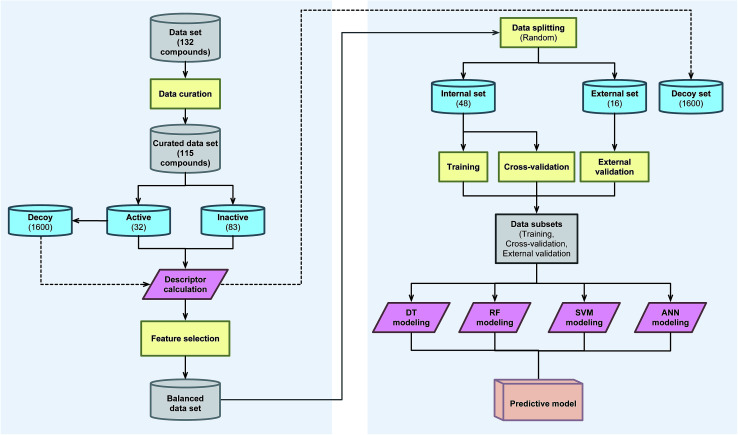
Workflow of CSAR modeling for investigating anti-sickling activity.

A schematic summary of the CSAR modeling process performed in this study is provided in Fig. 2.

### 2.1 Data collection

The compounds with anti-sickling activity used in this study were compiled from the literature,^14–20^ which afforded an initial set of 132 compounds. The removal of redundant compounds resulted in a final set of 115 compounds. The compounds were treated with the CSAR curation workflow as described by Fourches *et al.*^21^ ChemAxon Standardizer was utilized using the same protocol from our previous study.^22^ The anti-sickling activity is represented as a solubility ratio which is summarized below:1
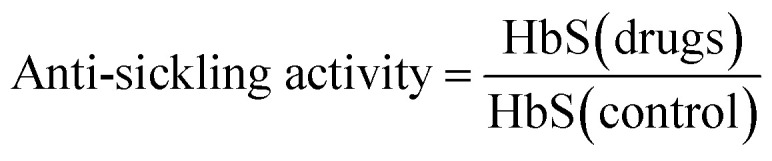
where HbS (drugs) and HbS (control) denotes the presence and absence of drugs in a solution of HbS. Solubility ratios greater than 1.06 were estimated as necessary for decreasing the clinical severity of sickle cell disease. Therefore, the compounds were classified into two types, consisting of 32 active compounds (solubility ratios of ≥1.06) and 83 inactive compounds (solubility ratios of <1.06). Moreover, a set of 1600 decoy molecules was generated from active compounds using DUD-E and treated as inactive compounds.^23^

### 2.2 Molecular descriptors

Molecular descriptors can be defined as the quantitative and/or qualitative description of molecules of interest in terms of their constitution, connectivity and physicochemical properties.^24,25^ They are of prime importance for quantitative structure–activity relationship (QSAR) studies.^26^ Molecular descriptors can be easily calculated from GUI-based software such as Dragon,^27,28^ PaDEL-Descriptor software,^29^ QuBiLS-MIDAS,^30^ QuBiLS-MAS^31^ and CODESSA;^32^ they can be derived programmatically *via* R or Python environments using packages/modules such as ChemoPy,^33^ PyDPI,^34^ RDKit^35^ and rcdk;^36^ and they can be obtained *via* the internet using webservers such as BioTriangle^37^ and ChemDes.^38^

Fingerprint descriptors provide descriptions of the constituting substructures inherently present in a molecule. This study makes use of the PaDEL-Descriptor software^29^ for computing several fingerprint classes. Until now, the current version of PaDEL has provided 1875 descriptors, consisting of 1444 1D and 2D descriptors and 431 3D descriptors, and 12 types of fingerprint (a total of 16 092 bits). In this study, we employed 12 types of fingerprint for describing the structural features of the investigated compounds as summarized in Table 1. Three of the twelve fingerprint classes pertain to the frequency count of the substructures presented in the investigated compounds (*i.e.* they contain the suffix count in the name of the fingerprint class, such as the substructure fingerprint count), while the remaining nine classes consider only the presence/absence of substructures or fingerprint bits in the investigated compounds.

**Table 1:** Summary of 12 sets of fingerprint descriptors

Fingerprint	Number	Descriptors	Ref.
CDK	1024	Fingerprint with a length of 1024 and a search depth of 8	39
CDK extended	1024	Extends CDK with additional bits describing ring features	39
CDK graph only	1024	Special version of CDK that does not account for bond orders	39
E-state	79	Electrotopological state for the electronic and topological characterization of atoms	40
MACCS	116	Binary representation of the chemical substructure by MACCS keys	41
PubChem	881	Binary representation of the PubChem fingerprint	42
Substructure	307	Presence of SMARTS patterns for functional group classification	43
Substructure count	307	Count of SMARTS Patterns for functional group classification	43
Klekota–Roth	4860	Presence of chemical substructures that enrich biological activity	44
Klekota–Roth count	4860	Count of chemical substructures that enrich biological activity	44
2D atom pairs	780	Presence of atom pairs at various topological distances	45
2D atom pair count	780	Count of atom pairs at various topological distances	45

### 2.3 Data filtering

Constant and near constant variables were employed to initially select fingerprint descriptors from a large data set of twelve fingerprint descriptor sets, which not only adds complexity but could potentially give rise to bias in the model. The constant of each fingerprint descriptor and bioactivity (anti-sickling) were calculated using a standard deviation (SD) of 0.1 as a cut-off value. The fingerprint descriptors with SD values greater than 0.1 were selected for further analysis. The numbers of descriptors after filteration are shown in Table 2, *i.e.* CDK (885-dimensional), CDK extended (892-dimensional), CDK graph only (441-dimensional), E-state (18-dimensional), MACCS (103-dimensional), PubChem (299-dimensional), substructure (38-dimensional), substructure count (45-dimensional), Klekota–Roth (340-dimensional), Klekota–Roth count (366-dimensional), 2D atom pairs (133-dimensional) and 2D atom pair count (167-dimensional).

**Table 2:** Performance summary of CSAR models for predicting anti-sickling agents

Descriptor class	*N*	Training set				5-Fold CV set				External set				Decoy
		Ac	Sn	Sp	MCC	Ac	Sn	Sp	MCC	Ac	Sn	Sp	MCC	
CDK	885	99.81 ± 0.67	99.92 ± 0.56	99.72 ± 1.15	1.00 ± 0.01	79.25 ± 5.91	78.06 ± 6.29	81.08 ± 6.78	0.59 ± 0.12	81.06 ± 10.77	80.50 ± 12.84	84.26 ± 12.83	0.63 ± 0.22	87.00 ± 3.42
CDK extended	892	99.88 ± 0.49	99.88 ± 0.68	99.88 ± 0.68	1.00 ± 0.01	79.71 ± 5.57	79.48 ± 6.41	80.43 ± 6.11	0.60 ± 0.11	80.19 ± 9.36	80.75 ± 10.95	82.50 ± 11.69	0.62 ± 0.18	87.59 ± 3.41
CDK graph only	441	96.52 ± 2.48	95.93 ± 3.51	97.29 ± 2.58	0.93 ± 0.05	77.92 ± 5.37	77.08 ± 6.03	79.38 ± 6.10	0.56 ± 0.11	77.63 ± 11.39	77.80 ± 13.23	79.89 ± 12.93	0.56 ± 0.23	84.71 ± 2.99
E-state	18	90.69 ± 3.01	90.28 ± 4.74	91.56 ± 3.69	0.82 ± 0.06	80.44 ± 6.69	79.06 ± 7.84	82.64 ± 6.51	0.61 ± 0.13	82.13 ± 8.62	81.12 ± 10.91	86.25 ± 11.03	0.66 ± 0.17	84.90 ± 2.40
MACCS	103	97.23 ± 2.02	98.17 ± 2.50	96.53 ± 3.30	0.95 ± 0.04	77.31 ± 5.91	77.21 ± 6.49	77.84 ± 6.24	0.55 ± 0.12	79.19 ± 9.31	80.47 ± 11.31	80.32 ± 10.88	0.60 ± 0.19	85.77 ± 4.13
PubChem	299	97.10 ± 2.48	97.06 ± 3.31	97.30 ± 2.78	0.94 ± 0.05	79.63 ± 4.75	78.10 ± 5.70	81.84 ± 5.02	0.60 ± 0.09	78.75 ± 9.52	77.79 ± 11.44	83.01 ± 11.91	0.59 ± 0.19	84.79 ± 3.00
Substructure	38	92.75 ± 3.14	95.30 ± 4.18	90.78 ± 3.93	0.86 ± 0.06	80.96 ± 5.26	81.68 ± 6.10	80.79 ± 5.75	0.62 ± 0.10	81.56 ± 8.86	82.96 ± 11.35	82.81 ± 10.97	0.64 ± 0.18	88.13 ± 3.12
Substructure count	45	95.58 ± 2.80	98.52 ± 2.43	93.15 ± 4.02	0.91 ± 0.05	82.50 ± 5.05	83.57 ± 5.59	81.85 ± 5.53	0.65 ± 0.10	82.38 ± 8.99	84.82 ± 11.32	83.27 ± 11.29	0.66 ± 0.17	85.93 ± 3.29
Klekota–Roth	340	98.00 ± 1.95	98.80 ± 1.96	97.32 ± 2.86	0.96 ± 0.04	79.31 ± 6.00	79.54 ± 7.62	79.74 ± 5.66	0.59 ± 0.12	79.19 ± 9.44	81.84 ± 12.08	79.89 ± 11.67	0.60 ± 0.19	87.23 ± 3.22
Klekota–Roth count	366	98.88 ± 1.49	99.31 ± 1.52	98.53 ± 2.40	0.98 ± 0.03	78.33 ± 5.26	78.72 ± 6.47	78.63 ± 5.80	0.57 ± 0.11	78.81 ± 9.60	80.71 ± 13.37	80.88 ± 11.22	0.59 ± 0.19	87.72 ± 3.26
2D atom pairs	133	94.58 ± 3.45	94.94 ± 4.51	94.46 ± 3.56	0.89 ± 0.07	79.04 ± 4.80	79.04 ± 5.83	79.43 ± 4.78	0.58 ± 0.10	78.69 ± 10.20	78.57 ± 11.45	81.47 ± 12.54	0.59 ± 0.20	84.00 ± 3.10
2D atom pair count	167	99.19 ± 1.02	99.96 ± 0.40	98.48 ± 1.95	0.98 ± 0.02	78.00 ± 4.58	78.53 ± 5.72	77.89 ± 4.79	0.56 ± 0.09	77.63 ± 10.28	79.17 ± 12.19	78.96 ± 11.92	0.57 ± 0.20	88.00 ± 2.64

### 2.4 Data balancing

As noted in the previous section, the data set was highly imbalanced as the ratio of active to inactive compounds was 1 : 2.6. From a machine learning point of view, such an imbalanced data set has a tendency to cause classifiers to overfit, as well as to perform poorly on the minority class. To alleviate this data imbalance issue, an undersampling technique was applied by randomly selecting a subset of 32 inactive compounds from the initial set of 83 inactive compounds. After obtaining the balanced data set consisting of 32 active and 32 inactive compounds, the total set of 64 compounds was divided into two subsets using an 8 : 2 ratio, consisting of 48 compounds in the internal set (24 active and 24 inactive) and 16 compounds in the external set (8 active and 8 inactive).

### 2.5 Data set modelability

The modelability of the data set is essentially dependent on the underlying relation of the chemical structures and their observed bioactivity. In particular, two highly similar compounds with striking differences in their bioactivity (*i.e.* one compound in a pair affords favorable bioactivity while the other affords poor bioactivity), otherwise known as an activity cliff, would be detrimental for machine learning algorithms in their attempts to correlate structures with related levels of bioactivity. On the other hand, similar compounds with similar bioactivities (*i.e.* where both compounds in a compound pair provide the same bioactivity class) would contribute favorably to the modelability of the data set. This so-called modelability index (MODI) was introduced by Golbraikh *et al.*^46^ for the *a priori* estimation providing the feasibility of building robust predictive models for any given data set. This statistical metric can be computed as follows:

#### Step 1

For any given pair of compounds, *C*_*i*_ and *C*_*j*_ defined by an *m*-dimensional vector, the normalized Euclidean distance (*d_ij_*′) is computed as follows:2
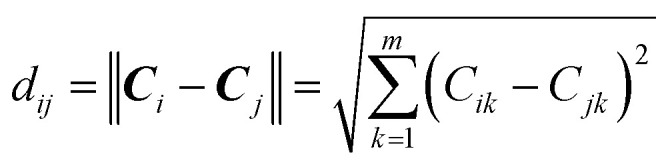
3
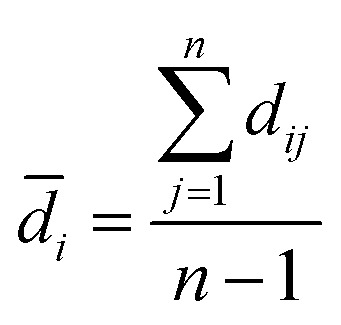
4
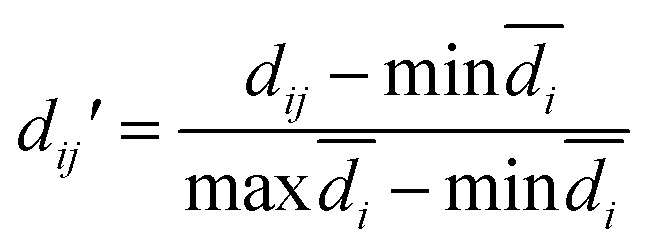
where *d*_*ij*_, *d̄*_*i*_ and *n* represent the distance scores between the two compounds, the mean Euclidean distance and the number of compounds, respectively.

#### Step 2

For each compound in a data set, the MODI can be computed by identifying its first nearest neighbor (*i.e.* the compound with the smallest Euclidean distance) belonging to the same or a different class as follows:5
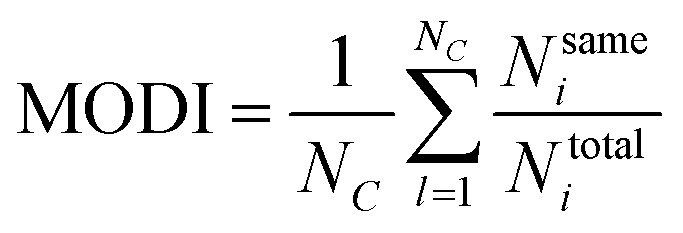
where *N*_*C*_ is the number of classes (*i.e. C* = 2 denotes active and inactive compounds), *N*^same^_*i*_ is the number of compounds of the *i*^th^ class that have their first nearest neighbors belonging to the same *i*^th^ class, and *N*^total^_*i*_ is the number of compounds belonging to the *i*^th^ class. A data set is considered to be modelable if the MODI index is greater than the threshold value of 0.65. The MODI index was computed using an in-house developed R code.

### 2.6 Statistical analysis

Statistical analysis was performed to investigate the difference patterns, features and trends that are present in individual descriptors between bioactivity classes (*i.e.* active and inactive) using 6 descriptive statistical parameters, comprising the minimum (Min), first quartile (Q1), median, mean, third quartile (Q3) and maximum (Max) parameters. A box plot of descriptors was created using the R package ggplot2.^47^ The normal distribution of each descriptor was assessed using Kolmogorov–Smirno tests from the ks.test function in the R stats package. Practically, the parametric *t*-test is applicable if the data follows a normal distribution, whereas for a non-normal distribution the non-parametric approach, namely the Mann–Whitney *U* test, is recommended. Particularly, the wilcox.test function from the R stats package^48^ was used.

### 2.7 Multivariate analysis

For a CSAR model, its prediction performance will depend not only on compound descriptors but also on the predictor used. This study employs random forest (RF) as the classifier owing to its demonstrated success in previous models as well as its interpretability. RF is an ensemble classifier that produces a number of decision trees, using a randomly selected subset of training samples and variables. The classification starts at the root node in which the data set at the node is split according to the value of the descriptors that are selected, such that descriptors of different activities are predominantly moved to different branches. The classification is obtained by averaging the results of all trees by a majority vote from each tree.^49,50^ The RF classifier was generated using the randomForest R package.

To accurately predict the anti-sickling activities of the compounds, it is necessary to tune two parameters of the RF model, *i.e.* the number of trees used for constructing the RF classifier (*n*_tree_) and the number of random candidate features (*m*_try_). In this study, a 10-fold CV procedure was applied to tune the *n*_tree_ parameter from the range of *n*_tree_ ∈ {100, 200, …, 1000}, while the *m*_try_ parameter was estimated using the tuneRF function in the randomForest R package.^51^ In order to provide a better understanding of the anti-sickling activities of the compounds, informative descriptors were extracted from the RF model by means of its built-in feature importance estimator. In particular, the mean decrease of the Gini index (MDGI) was utilized to estimate the important descriptors, in which the descriptors with the largest MDGI values represent the most important features, as these descriptors contribute most to the prediction performance of the model.

### 2.8 Model validation

The balanced data set was then subjected to a 5-fold repeated cross-validation (5-fold repeated CV) scheme and external validation so as to assess the model intrapolation and extrapolation, respectively. Briefly, data splitting was continuously resampled for 100 iterations (*i.e.* the data was reshuffled and re-stratified before each round) where each data split iteration divides the data set into internal and external sets using the 80/20 split ratio. Subsequently, the internal set (consisting of 48 compounds) was subjected to 5-fold repeated CV in which the data was partitioned into 5 folds, where one fold was retained as the testing set while the remaining folds were used to train the model. This process was repeated iteratively until all folds had the chance to be retained as the testing set. The partitioned 5 folds were reshuffled three times in a repeated CV fashion. Moreover, external validation was also performed on the external set and the decoy data set in order to assess the extrapolation capability of the model on unknown data that has not been previously seen by the training model.

The prediction of anti-sickling activity is essentially a binary (two-class) classification problem, *i.e.* whether the bioactivity of the compound is active or inactive. For this kind of binary classification problem, the following set of metrics, *i.e.* accuracy (Ac), sensitivity (Sn), specificity (Sp) and the Matthew’s correlation coefficient (MCC), were used to evaluate the prediction performance:6
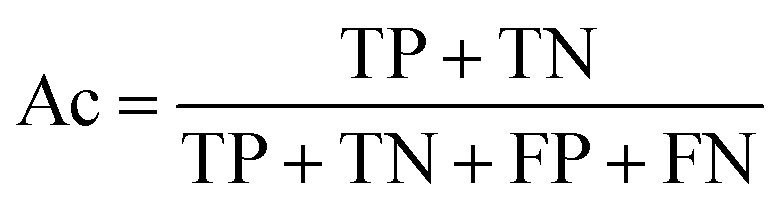
7
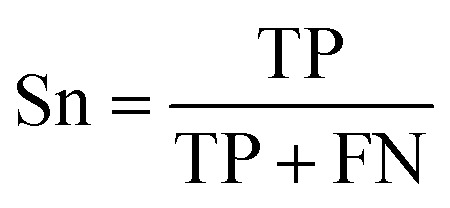
8
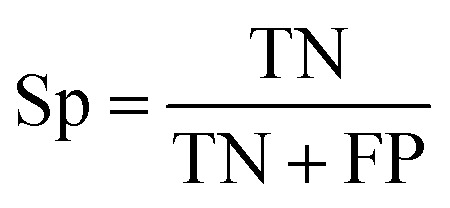
9
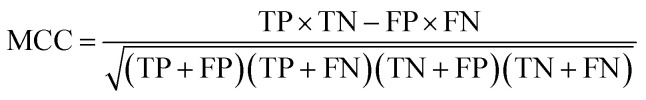
where TP, TN, FP and FN represent the instances of true positives, true negatives, false positives and false negatives, respectively. The value of MCC ranges from −1 to 1, in which an MCC of 1 indicates the best possible prediction scenario while an MCC of −1 indicates the worst possible prediction. On the other hand, an MCC of 0 is indicative of random prediction.

Furthermore, receiver operating characteristic (ROC) curves were plotted to show the predictive capability of our CSAR models using the pROC package in the R software.^52^ The ROC curve presents the model behaviour of the true positive rate (sensitivity) against the false positive rate (1-specificity) in a visual way. The area under the ROC curve (AUC) was calculated to quantitatively and objectively measure the performance of the proposed CSAR models. A random classifier has an area under the curve of 0.5, while the AUC for a perfect classifier is equal to 1.

### 2.9 Applicability domain analysis

Applicability domain (AD) analysis allows the definition of chemical space boundaries in which the classification model can be reliably used to predict the putative bioactivity of the investigated compounds.^24,53^ In particular, AD allows the relative estimation of the feasibility of predictions made on query compounds on the basis of how similar they are to the compounds used to train the model. There are several approaches that have been proposed to assess the AD of compounds.^54^ Of these approaches, the principal component analysis (PCA) bounding box is an intuitive approach which is based on the conversion of the original data into a new orthogonal coordinate system that also corrects for correlations amongst the descriptors. Newly formed axes are defined as PCs presenting the maximum variance of the investigated compounds in the data set. The AD of the classification model presented herein is represented by the PCA scores plot in which the boundary spanned by compounds from the training set is considered to be the AD of the model. Thus, if compounds from the testing set are found to fall within this defined boundary, they are also considered to be within the model’s AD, and *vice versa* (*i.e.* compounds from the testing set located outside the boundary of the training set space would be expected to be outside the model’s coverage).

### 2.10 Reproducible research

To support the reproducibility of the constructed CSAR models as described in this study, all R codes and associated input files (*e.g.* fingerprint descriptors, SMILES notations, biological activity, *etc.*) used to create the results, figures and tables are publicly available on GitHub at https://github.com/chaninlab/anti-sickling/.

## 3 Results and discussion

### 3.1 Chemical space of the anti-sickling agents

**Fig. 3:**
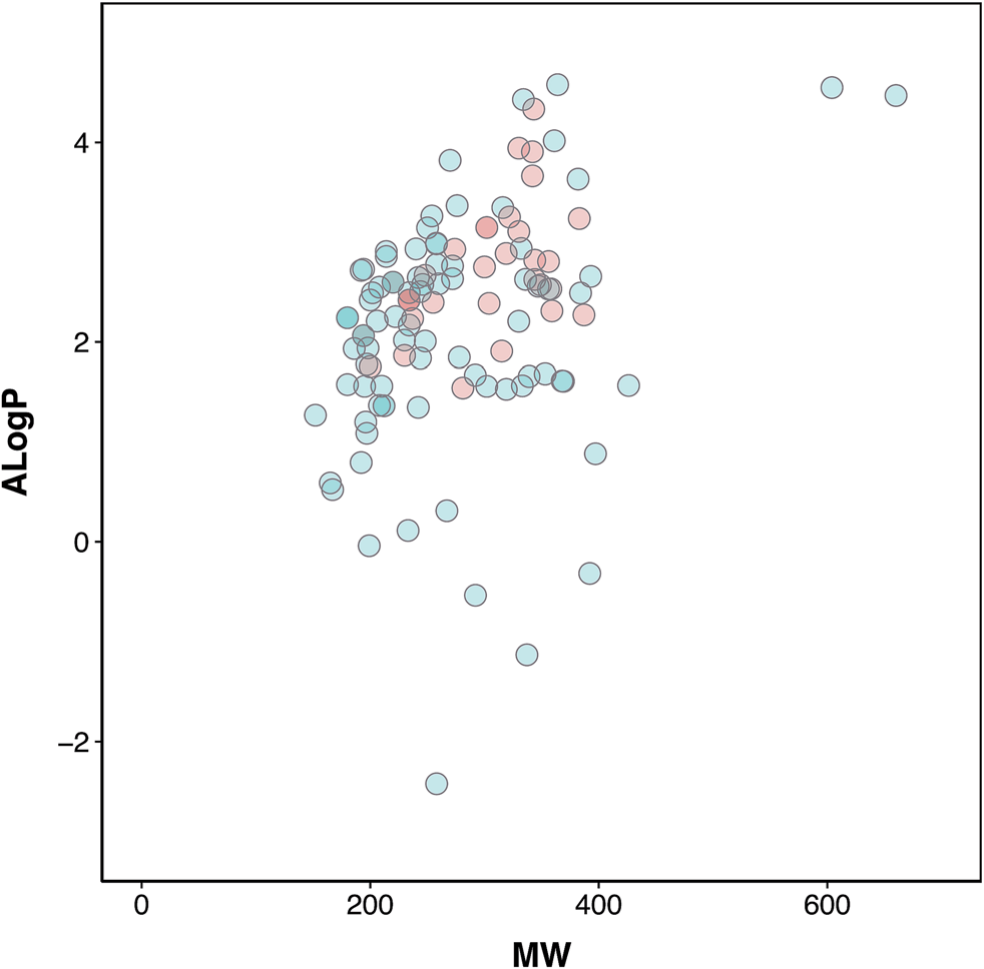
Chemical space of the anti-sickling agents. Actives and inactives are shown in red and green, respectively.

**Fig. 4:**
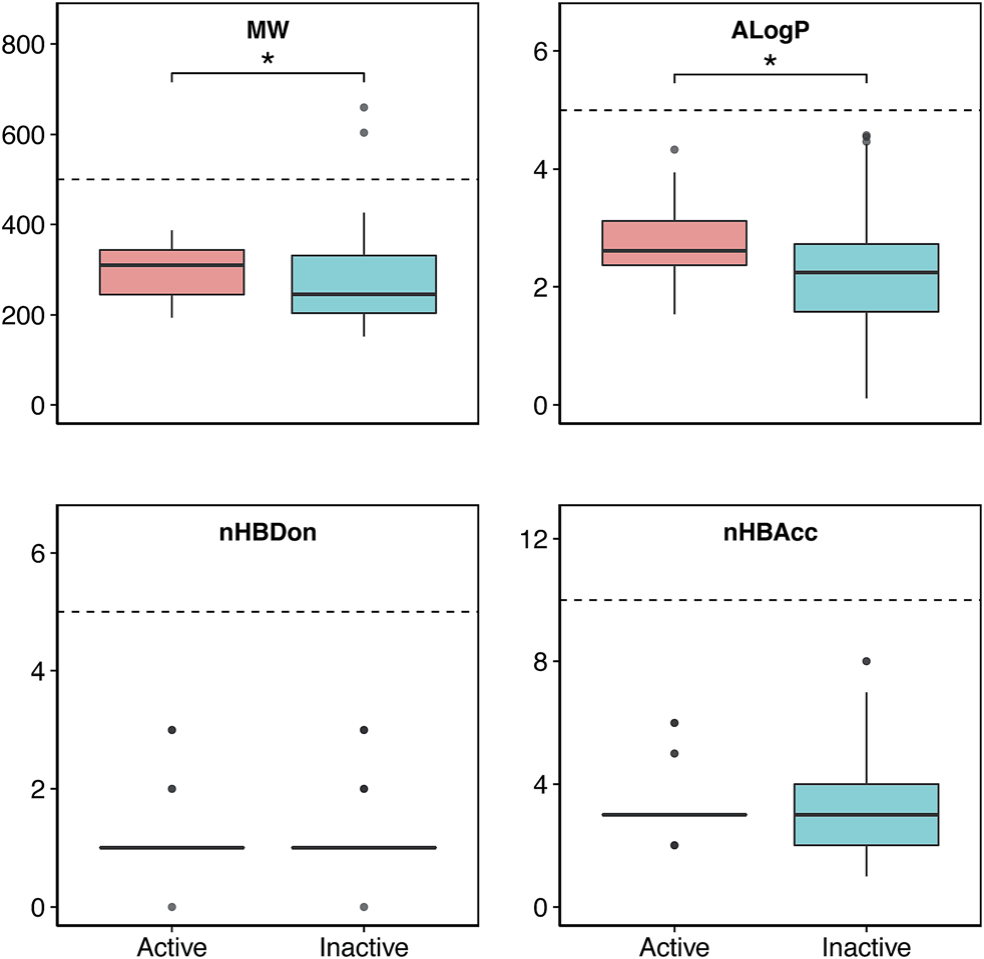
Box plot of the anti-sickling agents using Lipinski’s rule-of-five descriptors. Asterisks (*) denote significance at *p* ≤ 0.05.

Chemical space analysis was performed in order to explore the general characteristics of the active *versus* inactive classes of anti-sickling agents *via* the use of Lipinski’s rule-of-five descriptors. In particular, Lipinski’s rule-of-five descriptors are a refinement of drug-likeness used to predict whether a chemical compound will exhibit pharmacological or biological activity as an orally active drug in humans, based on the observation that most medications are relatively large-sized lipophilic molecules, comprising the molecular weight (MW), Ghose–Crippen–Viswanadhan octanol–water partition coefficient (*A*log*P*), number of hydrogen bond donors (nHBDon) and number of hydrogen bond acceptors (nHBAcc).^55^ The MW represents the mass of a compound, typically used for obtaining interpretations and calculations. Furthermore, the appropriate size of a compound is important for its passage *via* the lipid membrane. *A*log*P* is a well-known measure of molecular hydrophobicity (also known as lipophilicity), which is used for calculating the membrane penetration and permeability of compounds. nHBDon and nHBAcc are used to measure hydrogen bonding capacity. A visualization of the chemical space of *A*log*P* as a function of MW is shown in (Fig. 3). A dense distribution of anti-sickling compounds was observed within the MW range of approximately 200–400 Da and within the *A*log*P* range of approximately −2.5 to 4. In addition, a visualization of the overall distribution of the data values of Lipinski’s descriptors is shown as a box plot in (Fig. 4). It can be seen that most compounds follow the criteria of Lipinski’s rule where the MW is less than 500, except for 2 compounds (57 and 58). Furthermore, the *A*log*P* and nHBDon values are less than 5, and nHBAcc is less than 10.

Analysis of the box plots revealed that there were slight differences between the bioactivity classes (*i.e.* active and inactive) using Lipinski’s rule. In addition, the results of statistical analysis show significant differences in MW and *A*log*P* between the active and inactive compounds using the Mann–Whitney *U* test. The MWs of the active compounds were larger than those of the inactive compounds, which was observed from the mean value of the box plots. Similarly, the *A*log*P* values of the active compounds were greater than those of the inactive compounds, whereas the nHBDon and nHBAcc values of the active compounds were less than those of the inactive compounds.

### 3.2 CSAR modeling of the anti-sickling agents

Prior to initially establishing a prediction model, all activity cliffs must be detected, verified and treated using a score of the modelability of the data set or the MODI index. Herein, this index is used to identify the feasibility of obtaining a CSAR model for discriminating active compounds from inactive compounds. For binary data sets, if the MODI index is greater than 0.65, the data set should be reliable for classification modeling. Interestingly, 12 types of fingerprint met this criteria with their MODI index ranging from 0.70–0.84.

The interpretative predictive model is more useful for providing the basis of the biological and chemical properties of the anti-sickling agents. Herein, a CSAR model based on RF is presented for discriminating between the active and inactive compounds of anti-sickling agents. Table 2 lists the results from 100 independent runs for the RF model with twelve different types of fingerprint over an internal validation test, 5-fold CV and an external validation test. Furthermore, the decoy set was used to assess the abilities of the CSAR models to accurately predict inactive compounds. From Table 2, it was found that the best averaged values of Ac = 80.48 ± 5.46% and MCC = 0.61 ± 0.11, as evaluated by a 5-fold CV procedure, were achieved using the substructure count fingerprint descriptor. Meanwhile, the E-state and substructure fingerprints performed well, with the second and third highest averaged values of Ac/MCC of 79.88 ± 7.19%/0.60 ± 0.14 and 79.10 ± 6.87%/0.58 ± 0.14, respectively. Interestingly, as for the external validation test, the substructure count fingerprint was also found to outperform other descriptors in terms of their average values of Ac = 82.38 ± 8.99%, Sp = 84.82 ± 11.32%, Sn = 83.27 ± 11.29% and MCC = 0.66 ± 0.17. However, this descriptor provided a moderate Ac value of 85.93 ± 3.29% on the decoy data set, and it was comparable to the substructure that had the best Ac value of 88.13 ± 3.12%. Considering the results from 5-fold CV and the external validation tests, it can thus be seen that the substructure count was superior to other fingerprint classes.

### 3.3 Applicability domain

**Fig. 5:**
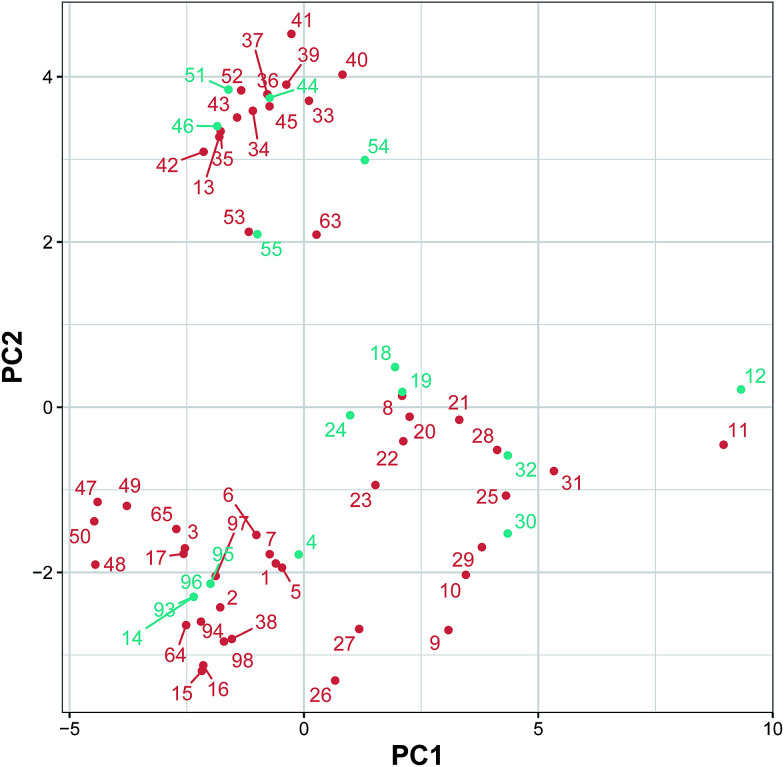
The applicability domain as analyzed using the PCA bounding box approach.

Fig. 5 shows the AD of the classification model built using the substructure count as estimated using the PCA bounding box. The undersampling technique was applied using the Kennard–Stone algorithm to select a subset of 32 inactive compounds from the initial set of inactive compounds for balancing. Afterwards, the total set of 64 compounds was split into two sets, consisting of training (80%) and testing (20%) sets, using the Kennard–Stone algorithm. The training and testing sets were subjected to PCA analysis and PCA bounding box plots were constructed for AD analysis. It can be observed that the compounds in the testing set were nearly located at the boundaries of the compounds in the training set, thereby suggesting a well-defined AD for the CSAR model.

### 3.4 Mechanistic interpretation of feature importance

The analysis of feature importance can provide a better understanding of the mechanistic details governing anti-sickling activity. In order to select informative descriptors on substructure counts, this study utilized the RF model because of its built-in ability of feature importance estimation and its great prediction performance, as discussed above. Generally, two measures were used to rank the important features, namely the mean decrease of the Gini index and the mean decrease of the accuracy. Since Calle and Urrea^56^ reported that the Gini index had more robust results compared to those from the accuracy, we utilized the mean decrease of the Gini index to rank the importance of the substructure count descriptors.

**Fig. 6:**
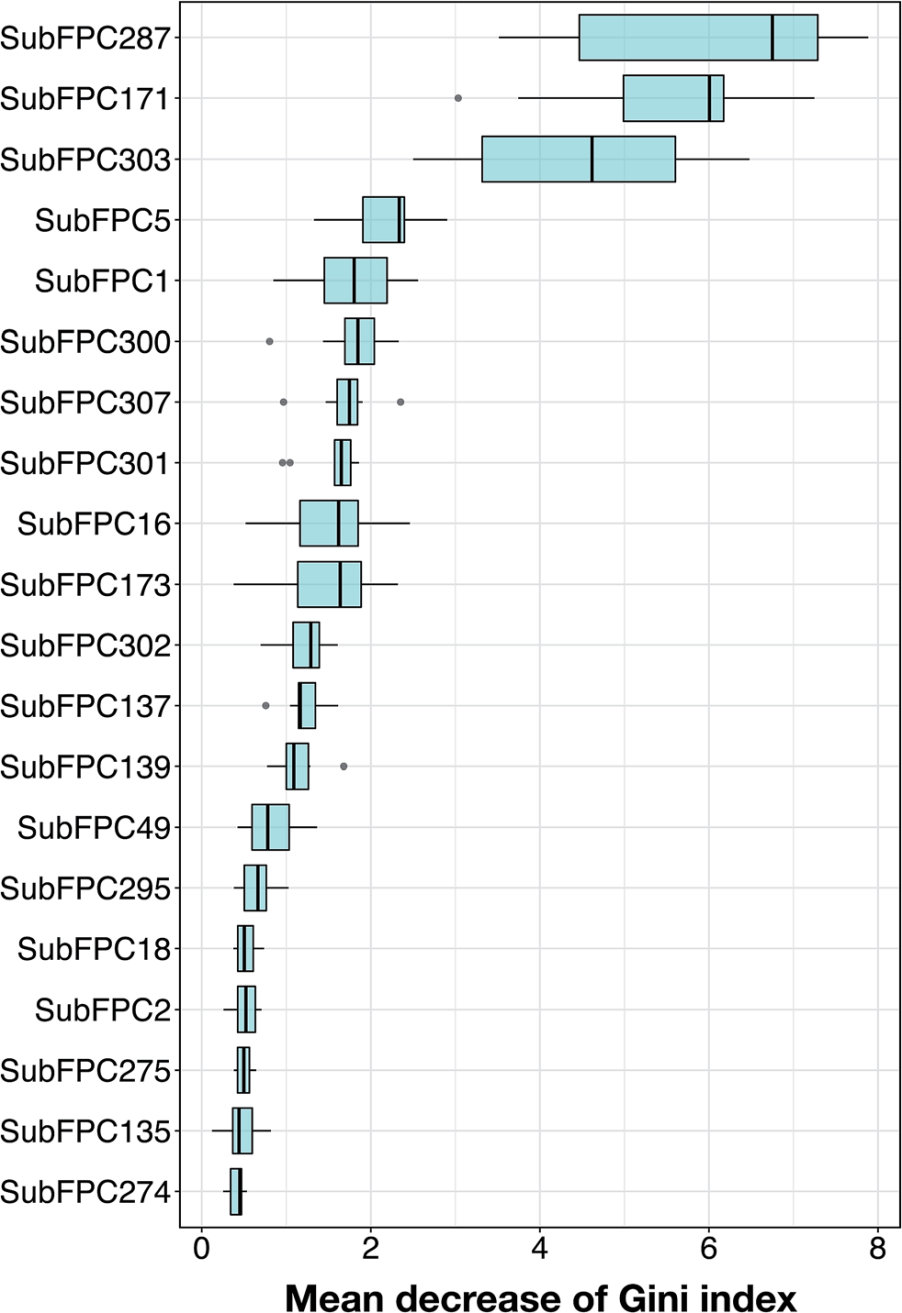
Descriptor importance of the substructure count fingerprints ranked by the mean decrease of Gini index.

As suggested previously,^49,50^ the *m*_try_ parameter could be obtained from the square root of the total number of features or by using the default value of *m*_try_ = 11. In this study, 10 RF models were constructed by varying the *m*_try_ parameter setting from 2 to 20 (*m*_try_ ∈ {2,3,5,7,9,11,13,15,17,19,20}) and fixing the *m*_tree_ parameter at 100. The use of multiple RF models might increase the reliability of the estimation of informative features. The descriptor importance for the substructure count, ranked by the mean decrease in the Gini index, is shown in Fig. 6, and detailed information for the 20 top-ranked informative descriptors is described in Table 3. The descriptor with the largest value of MDGI is the most important.

**Table 3:** List of the top substructure fingerprints and their descriptions

Ranking	Fingerprints	Description
1	SubFP287	Conjugated double bond
2	SubFP171	Aryl chloride
3	SubFP303	Michael acceptor
4	SubFP5	Alkene
5	SubFP1	Primary carbon
6	SubFP300	1,3-Tautomerizable
7	SubFP307	Chiral center specified
8	SubFP301	1,5-Tautomerizable
9	SubFP16	Dialkylether
10	SubFP173	Arylbromide
11	SubFP302	Rotatable bond
12	SubFP137	Vinylogous ester
13	SubFP139	Vinylogous halide
14	SubFP49	Ketone
15	SubFP295	C ONS bond
16	SubFP18	Alkylarylether
17	SubFP2	Secondary carbon
18	SubFP275	Heterocyclic
19	SubFP135	Vinylogous carbonyl
20	SubFP274	Aromatic

**Fig. 7:**
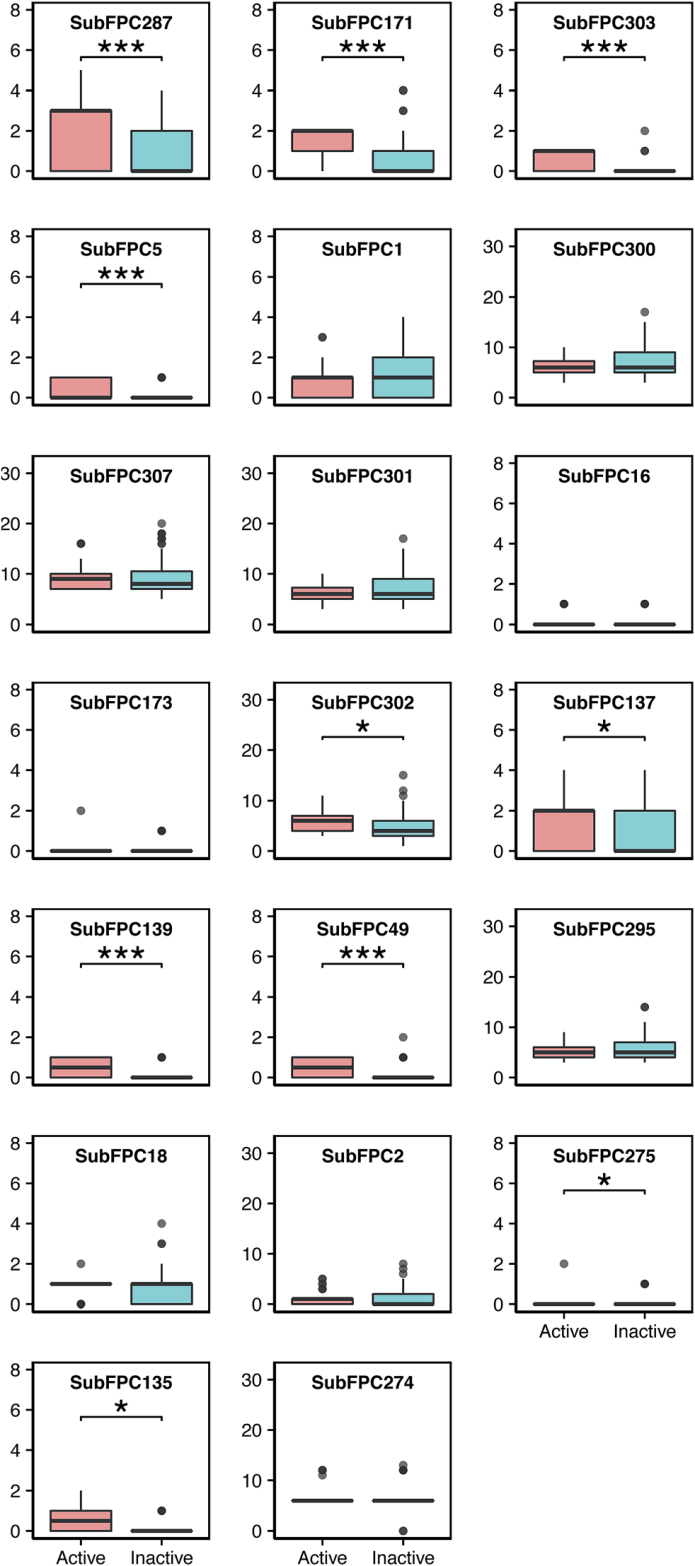
Box plots of anti-sickling agents using importance substructure fingerprints. A single asterisk (*) denotes significance at *p* ≤ 0.05, double asterisks (**) denote significance at *p* ≤ 0.001 and triple asterisks (***) denote significance at *p* ≤ 0.0001.

A further analysis was performed on each of these features by visualizing the prevalence of their functional moieties in the active *versus* inactive classes by means of a box plot, as shown in Fig. 7. The results showed that 10 out of the 20 top-ranked informative descriptors showed significant differences between the active and inactive compounds using the Mann–Whitney *U* test. It could be stated that these informative descriptors are beneficial for providing information on the different characteristics of the active and inactive compounds. Notably, these significant SubFPCs can be divided into three groups, encompassing compounds with aromaticity/conjugation, compounds with the carbonyl group moiety and miscellaneous compounds.

Interestingly, three out of the ten significant SubFPCs (SubFPC287, SubFPC171 and SubFPC5) belong to the general class of compounds with aromaticity/conjugation. The most important feature was SubFPC287, with an average Gini index value of 5.96, denoting the alternation of single and double bonds. This descriptor is commonly known as conjugation, in which the molecule contributes to a more stable structure due to the delocalization of charge through resonance and hybridization energy.^57^ It was found that molecular conjugation is more prevalent in the active class compared to the inactive class. We observed that 23 out of 32 active compounds and 28 out of 83 inactive compounds possessed this property. Moreover, we also found that SubFPC5 is more prevalent in the active compounds, ranking fourth with an average Gini index value of 2.18, corresponding to the alkene in SubFPC287. The second important feature was SubFPC171, with an average Gini index value of 5.56. SubFPC171 is essentially an aromatic ring with an attached chloride atom, known as an aryl chloride. This moiety has been demonstrated to be thermally stable as well as being capable of exhibiting nucleophilicity, owing to its inherent electron-rich properties.^58^ Interestingly, SubFPC171 can be found predominantly in almost all active compounds, except for a few (2d, 3d, 20a, 21a, 17b and 3c).

It was found that 27 out of 32 active compounds and 23 out of 83 inactive compounds possessed this moiety. Furthermore, the results of the analysis of the different types of aryl chloride in the active class revealed that compounds 22a, 6c and 18c contain monochlorobenzene, while the remaining compounds contain the dichlorobenzene ring. This feature is related to the increase in activity of the compounds for binding these moieties.^15^

Four out of the ten significant SubFPCs (SubFPC137, SubFPC139, SubFPC49 and SubFPC135) belonged to the general class of compounds containing the carbonyl group moiety. A carbonyl group is a carbon atom double-bonded to an oxygen atom. In particular, this moiety is polar due to the electronegativity of the oxygen atom, and it is more soluble in water as it forms H-bonds. The twelfth important feature was SubFPC137, with an average Gini index value of 1.21 denoting a vinylogous ester (R–O–CH=CHCOR′), which is an ester that is relatively similar to a double bond, containing the carbonyl and ethoxy groups. The ethoxy group is known as an ethyl phenyl ether, and it is much more similar to an ester than an ether due to the conjugation between the carbonyl group and the double bond. The resonance of this moiety is also similar to an ester, and therefore it presents a very unique electronic environment for the alkene group.^59^ This moiety can be seen conspicuously in the active class. Specifically, 19 out of 32 active compounds and 27 out of 83 inactive compounds contained this moiety. The thirteenth important feature was SubFPC139, with an average Gini index value of 1.14 denoting a vinylogous halide, which contains the carbonyl and halide groups. Interestingly, this SubFPC is correlated with SubFPC49, which has an average Gini index value of 0.83 denoting a ketone. We observed that these moieties were apparently found in the active class: 16 out of 32 active compounds possessed these moieties. Furthermore, the nineteenth important feature was SubFPC135, with an average Gini index value of 0.47 denoting the vinylogous carbonyl group, which consists of a carbonyl group and another atom (*e.g.* nitrogen, oxygen, sulfur or a halide). This moiety was obviously found in 24 out of 32 active compounds and 19 out of 83 inactive compounds.

Moreover, other significant SubFPCs are miscellaneous descriptors (*e.g.* SubFPC303, SubFPC302 and SubFPC275). The third important feature was SubFPC303, with an average Gini index value of 4.50 denoting a Michael acceptor, which is a conjugated system with an electron-withdrawing group as an electrophile and a resonance-stabilizing activating group, which stabilizes the anionic intermediate such as an acrylate ester, acrylonitrile, acrylamide, maleimide, alkyl methacrylate, cyanoacrylate or vinyl sulfone.^60^ It can be seen that 18 out of 32 active compounds contained a Michael acceptor whereas 3 out of 83 inactive compounds contained a Michael acceptor. The eleventh important feature was SubFPC302 with an average Gini index value of 1.21, denoting a rotatable bond. These are bonds which allow free rotation around themselves, defined as a single bond.^61^ This moiety was important for the determination of molecular flexibility. Notably, it was found that rotatable bonds were found in all of the compounds in the data set. In particular, they were highly prevalent in the active compounds. The eighteenth important feature was SubFPC275, with an average Gini index value of 0.50 denoting a heterocycle, which is a cyclic compound containing atoms of at least two different elements as members of its ring. It was observed that this moiety is more prevalent in the inactive class. Notably, 1 out of 32 active compounds (28a) and 20 out of 83 inactive compounds possessed this moiety. Therefore, the heterocyclic moiety may reduce anti-sickling activity.

### 3.5 Scaffold and substructure analysis

**Fig. 8:**
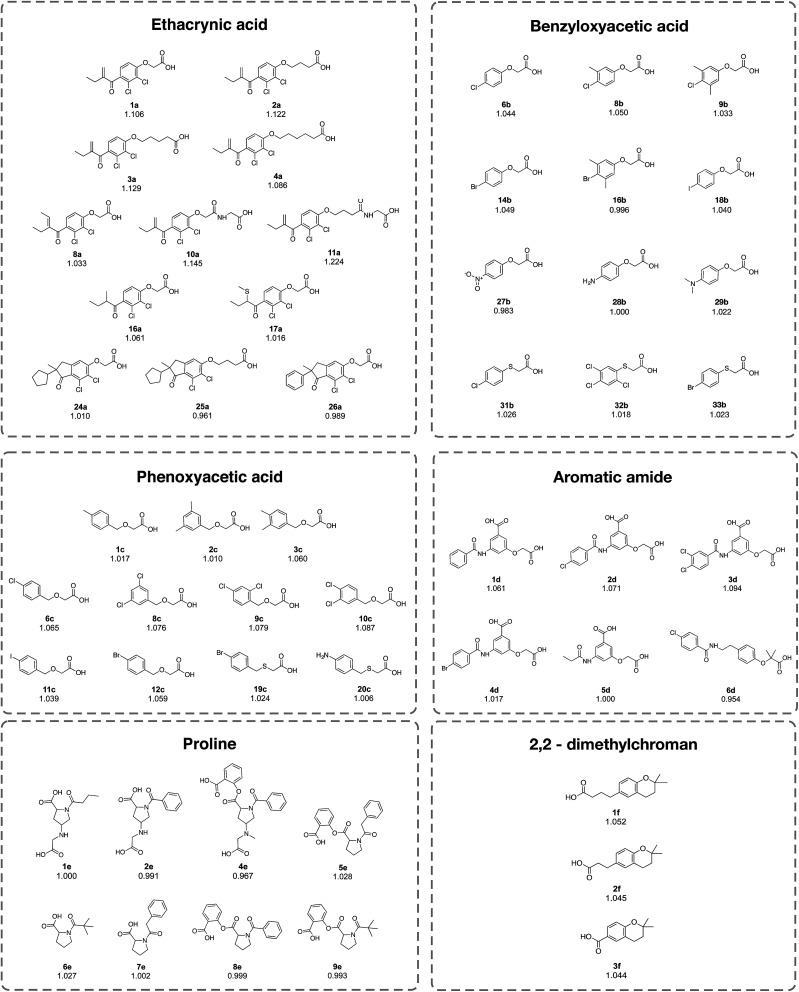
Chemical structures of the representative compounds as described in Table 4 from the analysis of the structure–activity relationship. It should be noted that the chemical structures of all compounds are provided in the ESI, Fig. 1–6.†

The investigated compounds were divided into 6 classes on the basis of their chemotypes, consisting of ethacrynic acid (ECA) analogs, benzyloxyacetic acid-based compounds, phenoxyacetic acid-based compounds, aromatic amide-based compounds, proline-based compounds and 2,2-dimethylchroman-based compounds (Table 4 and Fig. 8). Analysis of the structure–activity relationship (SAR) revealed that the length of the alkyl chain, as well as the functional moiety and substitutions on the benzene ring, may influence the anti-sickling activity of compounds.

Summary of the structure–activity relationship analysis as a function of the chemotypes and substructures

**Table:** 

Influential substructures	Chemotypes					
	Ethacrynic acid	Benzyloxyacetic acid	Phenoxyacetic acid	Aromatic amide	Proline	2,2-Dimethylchroman
Alkyl chain length	• Short alkyl chain > long alkyl chain 3a > 4a and 24a > 25a			• Long alkyl chain > short alkyl chain 7d > 5d	• Long alkyl chain > short alkyl chain 5e > 8e	• Long alkyl chain > short alkyl chain 1f > 2f > 3f
	• Long alkyl chain > short alkyl chain **2a** ≈ **3a** > 1a and 11a > 10a					
Functional moiety	• Cyclopentane > benzene 24a > 26a	• 2,3–Dihydrobenzo- furan > indane 21b > 20b	• Addition of benzene Central benzene: ↓activity 6c > 7c Peripheral benzene: ↑activity 16c > 15c	• Benzene > alkyl chain 1d > 5d	• Alkyl chain > benzene 1e > 2e	
	• Presence of vinyl moiety: ↑activity 1a > 8a and 1a > 16a				• CH_3_NO_2_ moiety: ↓activity 8e > 4e	
	• CH_3_S moiety: ↓activity 16a > 17a				• C_6_H_5_COO moiety: ↑activity 5e > 7e	
					• C_6_H_5_COO moiety: ↓activity 6e > 9e	
Substitutions on benzene		• Halogen atoms: Br > Cl > I 14b > 6b > 18b	• Halogen atoms: Cl > Br > I 6c > 12c > 11c	• Cl substitution: di-Cl > mono-Cl > without Cl 3d > 2d > 1d		
		• CH_3_ substitution: Mono-CH_3_ > di-CH_3_8b > 9b Without CH_3_ > di-CH_3_14b > 16b	• Cl substitution: di-Cl > mono-Cl 8c ≈ 9c ≈ 10c > 6c	• Halogen atoms: Cl > Br 2d > 4d		
			• CH_3_ substitution: Di-CH_3_ > mono-CH_3_3c ≈ 4c > 1c			
		• Nitrogen containing substitution: (CH_3_)_2_NH > NH_2_ > NO_2_29b > 28b > 27b

**Table:** 

	2-(Benzylthio)acetic acid	2-(Phenylthio)acetic acid
Substitutions on benzene	• Cl substitution: mono-Cl > tri-Cl 31b > 32b	• Br > NH_2_19c > 20c
	• Halogen atoms: Cl > Br 31b > 33b	

Compounds in the ECA class (1a–**31a**) exhibited the most potent anti-sickling activity when compared to the other chemotypes described herein. This is reflected by the highest solubility ratio in the range of 0.961 to 1.224. In particular, ECA was noted for its ability to cross the RBC membrane.^20^ The crucial chemical feature required for HbS binding was suggested to be the vinyl moiety and the substitution of halogen atoms on the benzene ring.^15,20^ Notably, compound 11a (solubility ratio = 1.224) was shown to be the most potent compound in the data set as it contains many significant SubFPCs present in the active class. Furthermore, the results showed that the length of the alkyl chain and the functional moiety may influence the anti-sickling activity of the ECA analogs. The cyclopentane moiety may enhance the anti-sickling activity of these compounds whereas the benzene ring leads to a decrease in the activity (*e.g.*24a > 26a). Moreover, the addition of the vinyl group may increase the anti-sickling activity (*e.g.*1a > 8a and 1a > 16a). On the other hand, the addition of the CH_3_S moiety may reduce the anti-sickling activity (*e.g.*16a > 17a).

The type and number of chemical moieties that are substituted on the benzene ring have been shown to influence the anti-sickling activity of the benzyloxyacetic acid analogs (1b–34b) and phenoxyacetic acid analogs. Mono-substitution with Br and Cl provided more potent activity compared to substitution with I in both classes of compounds (*i.e.* for benzyloxyacetic acid, Br > Cl > I = 14b > 6b > 18b and for phenoxyacetic acid, Cl > Br > I = 6c > 12c > 11c). This could be due to the large size of the I atom that may affect the access or interaction of compounds at the target site of action. A similar effect was also observed for aromatic amides, in which the chlorine analog (2d) was found to provide better activity than the bromide analog (4d). Likewise, the addition of a methyl CH_3_ group onto the benzene ring of the Cl and Br benzyloxyacetic acid derivatives may reduce the anti-sickling activity owing to an increase in bulkiness (*e.g.*8b > 9b and 14b > 16b). This finding is in accordance with a previous study^19^ demonstrating that the anti-sickling activity of compounds may not be enhanced by the insertion of methyl and polar groups. In contrast, multiple substitution on the benzene ring of the phenoxyacetic acid core structure led to enhanced activity of the compounds when compared to mono-substitution (*i.e.*8c ≈ 9c ≈ 10c > 6c and 3c ≈ 4c > 1c).

The same phenomenon was also observed for aromatic amide compounds (*i.e.* di-Cl > mono-Cl > without Cl = 3d > 2d > 1d). Moreover, the influence on the anti-sickling activity caused by the length of the substituted alkyl chain was exemplified for proline, aromatic amide and 2,2-dimethylchroman compounds, in which longer chain derivatives were shown to provide more potent activity than those with a shorter chain length (*i.e.*4d > 6d, 5e > 8e and 1f > 2f > 3f). Interestingly, the oxygen atom on the phenoxyacetic acid and benzyloxyacetic acid core structures may be required for the potent anti-sickling activity of these compounds. The replacement of the oxygen atom with a sulfur atom led to a decrease in activity, as found in the halogen derivatives of 2-(phenylthio)acetic acid (6b > 31b and 14b > 33b) and 2-(benzylthio)acetic acid (12c > 19c) derivatives.

### 3.6 Data-driven design of novel compounds

**Fig. 9:**
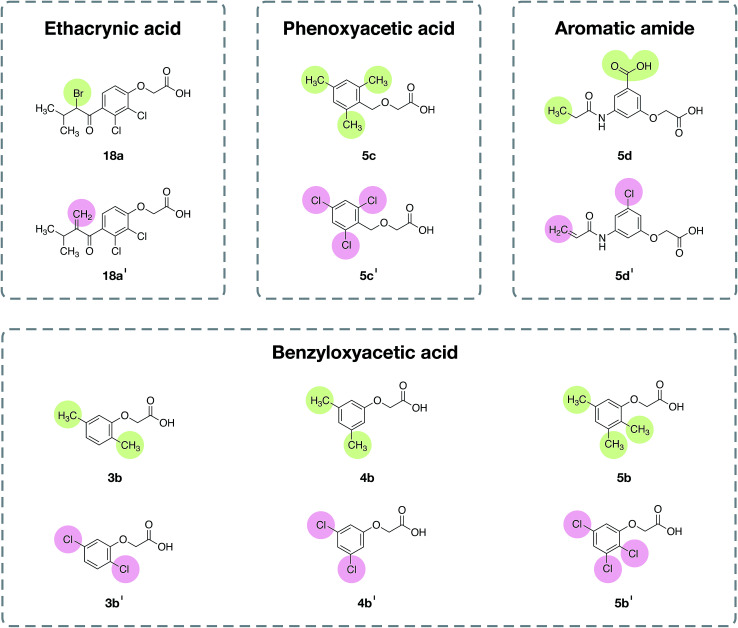
Chemical structures of the designed compounds. Six template compounds (the top row of each box) representing four chemotypes (ethacrynic acid, benzyloxyacetic acid, phenoxyacetic acid and aromatic amide) served as chemical starting points for designing novel analogs (the bottom row of each box). Green circles represent the original moieties of the template compounds and pink circles represent the replacement moieties of the designed compounds.

To apply the newfound knowledge from the feature importance and scaffold analyses, a set of compounds was designed by modifying existing compounds from the anti-sickling data set. The following criteria were considered for the selection of template compounds: (i) the selected compounds were labeled to show inactivity with a value less than 1.06, and (ii) selected compounds did not contain heterocyclic substructures that may cause a reduction in anti-sickling activity. These criteria led to the selection of four scaffolds (ECA, benzyloxyacetic acid, phenoxyacetic acid and aromatic amide). The designed compounds for each scaffold are shown in Fig. 9.

In the ECA scaffold class, compound 18a was modified by replacing the bromine atom with the vinyl group (18a′). Notably, the vinyl moiety was suggested to be an important feature for the ECA class, as mentioned in the scaffold and substructure analysis. Compounds 3b, 4b and 5b from the benzyloxyacetic acid class, as well as 5c from the phenoxyacetic acid class, were modified *via* the attachment of a methyl group to the Cl atom (3b′, 4b′ and 5b′ and 5c′) as mentioned in the feature importance (SubFPC171) and scaffold/substructure analysis. Compound 5d from the aromatic amide scaffold was modified by replacing the carboxyl group with a Cl atom (5d′). In spite of this, it was found that Cl modification alone was not sufficient to change the bioactivity. Thus, the vinyl moiety was selected to replace the terminal methyl group in the aromatic amide scaffold. The classification model was then applied to the set of designed compounds in order to predict their possible anti-sickling activity. It was found that the activity class of the evaluated compounds changed from the inactive class to the active class. Thus, the results indicated that the CSAR model as well as the scaffold and substructure analysis are useful for the compound design.

## 4 Conclusion

The hallmark of SCD is HbS polymerization, and the consequent conformation change of the RBCs to that of a sickle shape is associated with increased hemolysis. A lucrative therapeutic strategy for SCD is to employ small-molecule inhibitors for disrupting HbS polymerization. A total of 115 compounds were compiled from the literature and the resulting data set was balanced and used for model construction. Several classes of fingerprint descriptors and machine learning algorithms were evaluated for their ability to robustly predict anti-sickling activity. The results indicated that substructure fingerprints, together with the RF method, afforded the best performance while also affording an interpretable set of descriptors. As such, the origin of anti-sickling activity was deduced by rationalizing the contributions of important substructures as selected by the RF-derived Gini index. Feature analysis of the active compounds revealed the importance of aromaticity/conjugation (*i.e.* SubFPC287, SubFPC171 and SubFPC5, corresponding to a conjugated double bond, an aryl chloride and an alkene, respectively), carbonyl groups (*i.e.* SubFPC137, SubFPC139, SubFPC49 and SubFPC135, corresponding to a vinylogous ester, a vinylogous halide, a ketone and a vinylogous carbonyl, respectively) and miscellaneous groups (*e.g.* SubFPC303 and SubFPC302, corresponding to a Michael acceptor and a rotatable bond, respectively). Moreover, analysis of the structure–activity relationship revealed that the length of the alkyl chain and the substitution on the benzene ring may affect the anti-sickling activity of these compounds. Thus, the knowledge gained from this study serves as general guidelines for the data-driven design of potentially active anti-sickling agents.

## Conflicts of interest

There are no conflicts to declare.

## Supplementary Material

RA-008-C7RA12079F-s001
