# Correction: Privileged substructures for anti-sickling activity *via* cheminformatic analysis

**DOI:** 10.1039/c8ra90013b

**Published:** 2018-02-21

**Authors:** Chuleeporn Phanus-umporn, Watshara Shoombuatong, Veda Prachayasittikul, Nuttapat Anuwongcharoen, Chanin Nantasenamat

**Affiliations:** Center of Data Mining and Biomedical Informatics, Faculty of Medical Technology, Mahidol University Bangkok 10700 Thailand chanin.nan@mahidol.edu

## Abstract

Correction for ‘Privileged substructures for anti-sickling activity *via* cheminformatic analysis’ by Chuleeporn Phanus-umporn *et al.*, *RSC Adv.*, 2018, **8**, 5920–5935.

Errors were present in the published version of Fig. 2; the correct version is shown below:

**Fig. 2:**
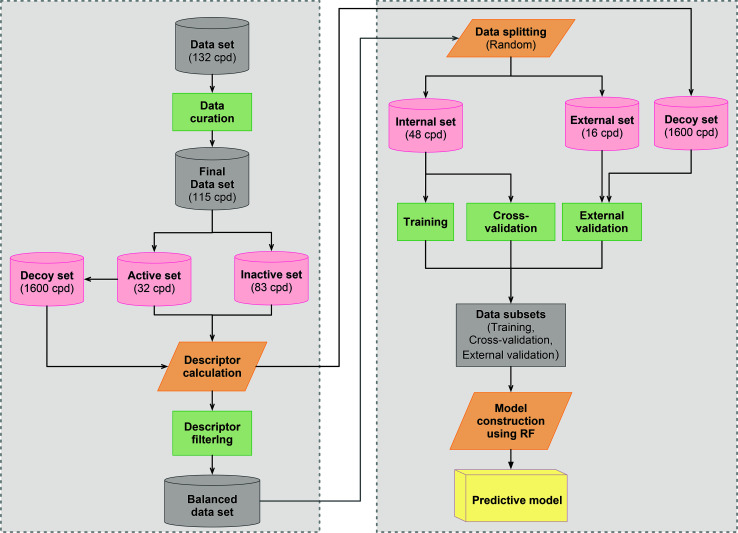
Workflow of CSAR modeling for investigating anti-sickling activity.

The Royal Society of Chemistry apologises for these errors and any consequent inconvenience to authors and readers.

## Supplementary Material

