# Supplementary material for: Privileged substructures for anti-sickling activity via cheminformatic analysis
Source: RSC Adv. 2018 Feb 7;8(11):5920–35. doi: 10.1039/c7ra12079f (PMC9078244; doi:10.1039/c7ra12079f)
Supplement: RA-008-C7RA12079F-s001 [file RA-008-C7RA12079F-s001.pdf]

Electronic Supplementary Material (ESI) for RSC Advances.
This journal is © The Royal Society of Chemistry 2018

Ethacrynic acid

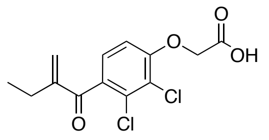

1a
1.106

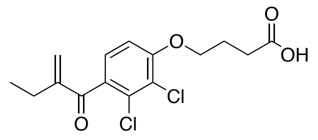

2a
1.122

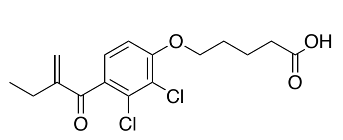

3a
1.129

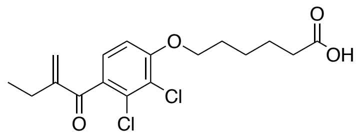

4a
1.086

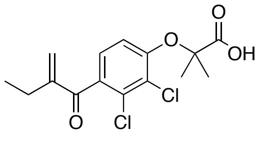

5a
1.094

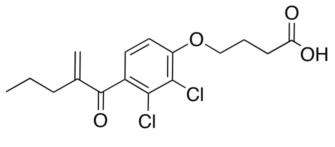

6a
1.115

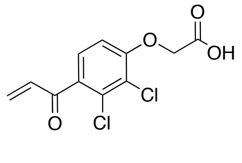

7a
1.060

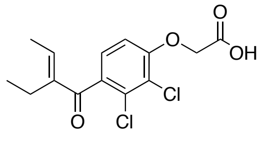

8a
1.033

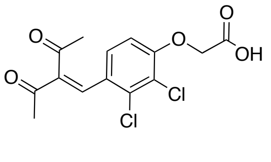

9a
1.010

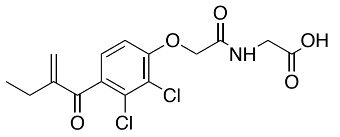

10a
1.145

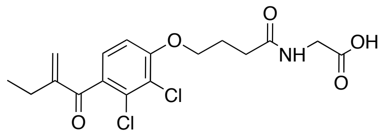

11a
1.224

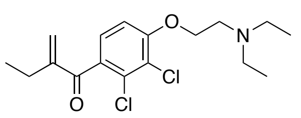

12a
1.077

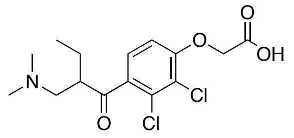

13a
1.018

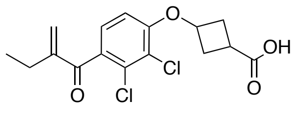

14a
1.127

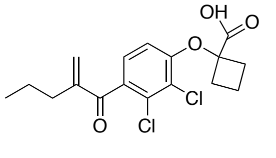

15a
1.085

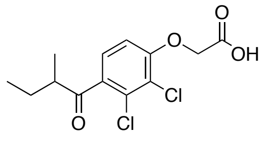

16a
1.061

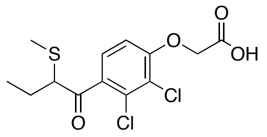

17a
1.016

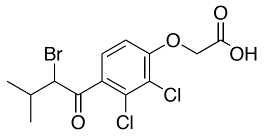

18a
0.966

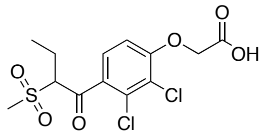

19a
1.029

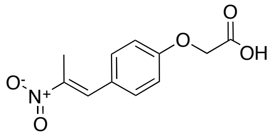

20a
1.076

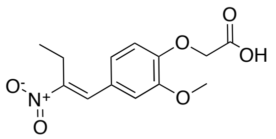

21a
1.128

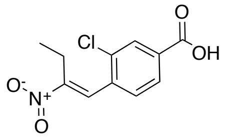

22a
1.100

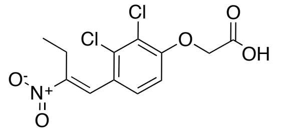

23a
1.086

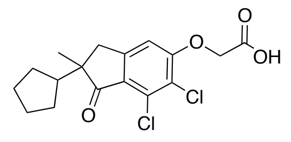

24a
1.010

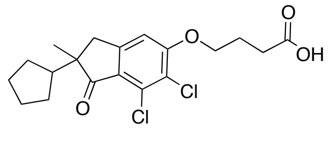

25a
0.961

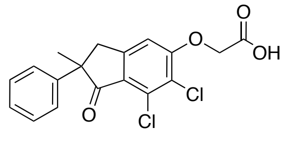

26a
0.989

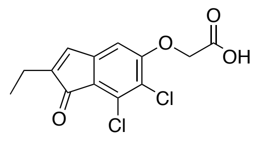

27a
1.090

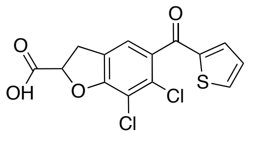

28a
1.076

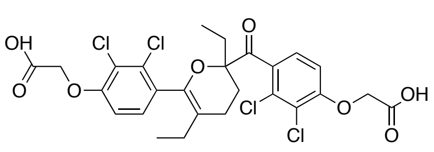

29a
0.993

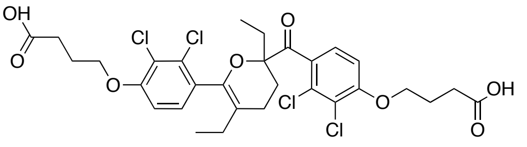

30a
1.059

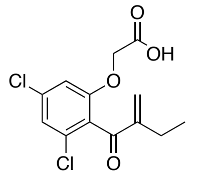

31a
1.080

Supplementary Fig. 1. Chemical structures of ethacrynic acid analogs and their anti-sickling activity (HbS ratio ranging from 0.961-1.224).

Benzyloxyacetic acid

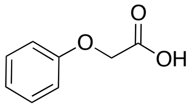

1b
1.017

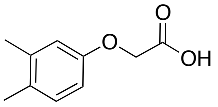

2b
1.027

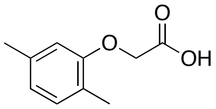

3b
1.031

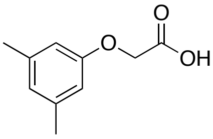

4b
1.035

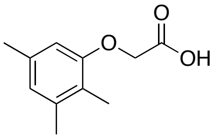

5b
1.035

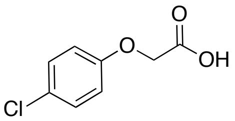

6b
1.044

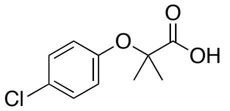

7b
1.018

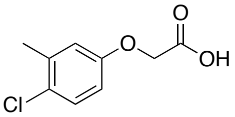

8b
1.050

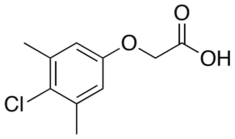

9b
1.033

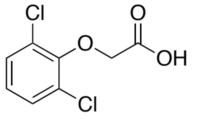

10b
1.025

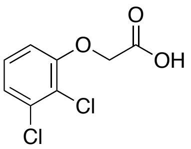

11b
1.025

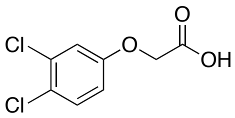

12b
1.061

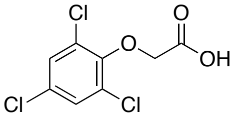

13b
1.057

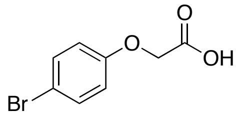

14b
1.049

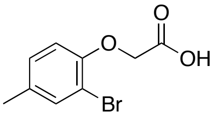

15b
1.031

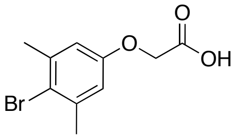

16b
0.996

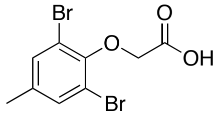

17b
1.077

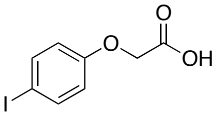

18b
1.040

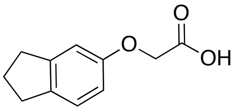

19b
1.054

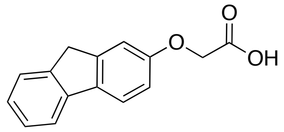

20b
1.029

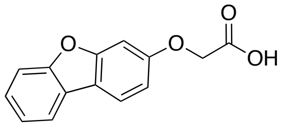

21b
1.033

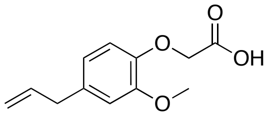

22b
1.033

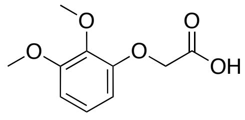

23b
1.017

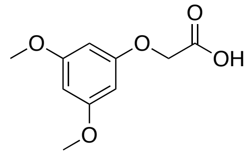

24b
1.030

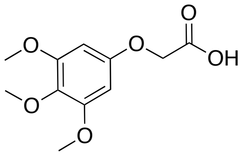

25b
0.987

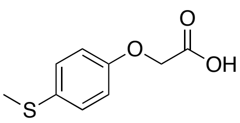

26b
1.035

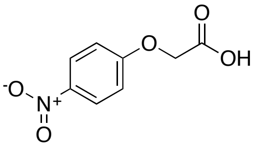

27b
0.983

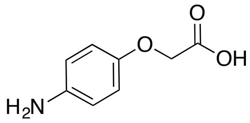

28b
1.000

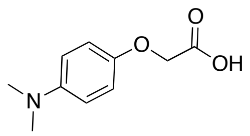

29b
1.022

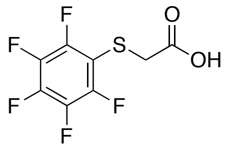

30b
1.025

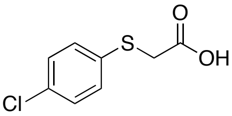

31b
1.026

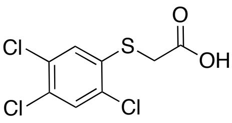

32b
1.018

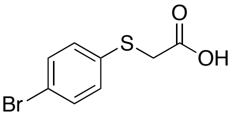

33b
1.023

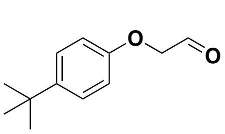

34b
1.004

Supplementary Fig. 2. Chemical structures of benzyloxyacetic acid analogs and their anti-sickling activity (HbS ratio ranging from 0.983-1.077).

Phenoxyacetic acid

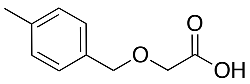

1c
1.017

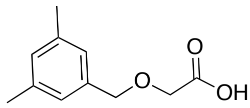

2c
1.010

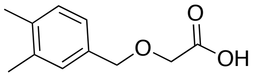

3c
1.060

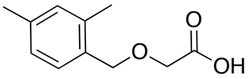

4c
1.050

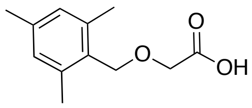

5c
1.031

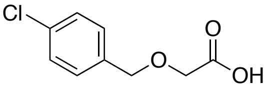

6c
1.065

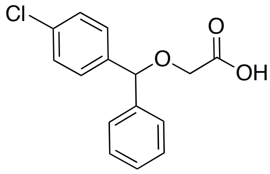

7c
1.002

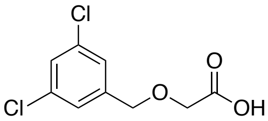

8c
1.076

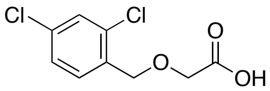

9c
1.079

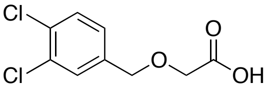

10c
1.087

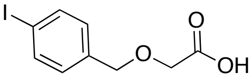

11c
1.039

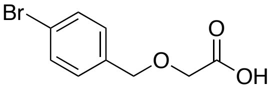

12c
1.059

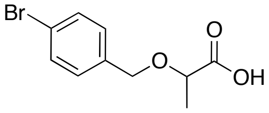

13c
1.048

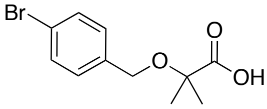

14c
1.048

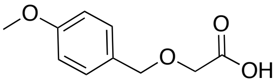

15c
0.996

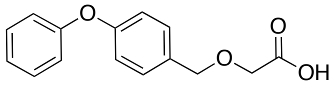

16c
1.034

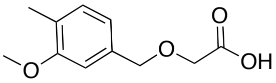

17c
1.018

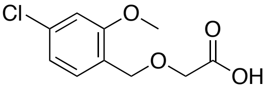

18c
1.062

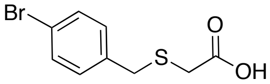

19c
1.024

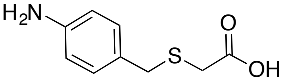

20c
1.006

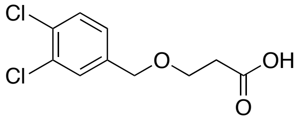

21c
1.062

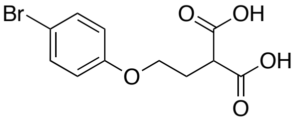

22c
1.057

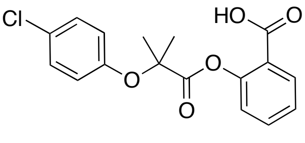

23c
1.022

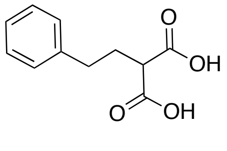

24c
1.007

Supplementary Fig. 3. Chemical structures of phenoxyacetic acid analogs and their anti-sickling activity (HbS ratio ranging from 0.996 to 1.087).

Aromatic amide

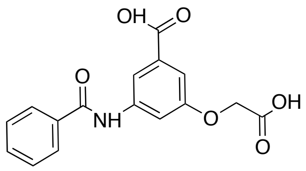

1d
1.061

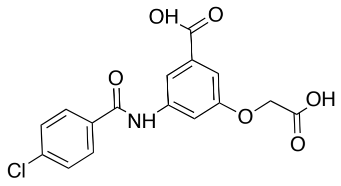

2d
1.071

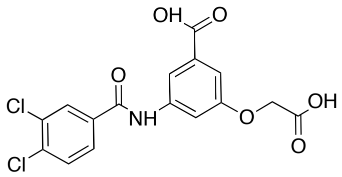

3d
1.094

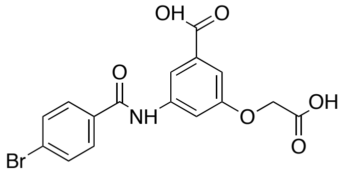

4d
1.017

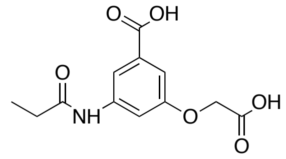

5d
1.000

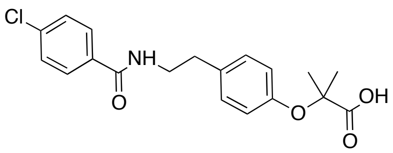

6d
0.954

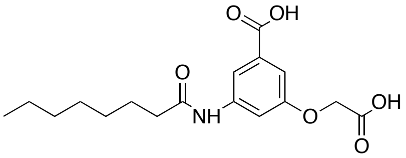

7d
1.046

Supplementary Fig. 4. Chemical structures of aromatic amide analogs and their anti-sickling activity (HbS ratio ranging from 0.954 to 1.094).

Proline

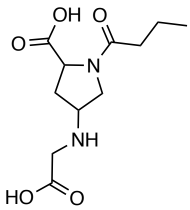

1e
1.000

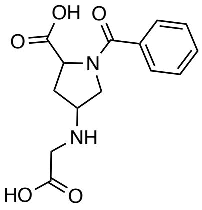

2e
0.991

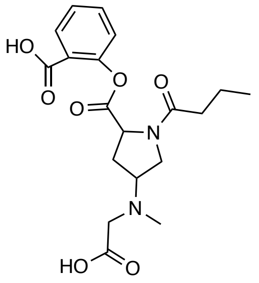

3e
0.965

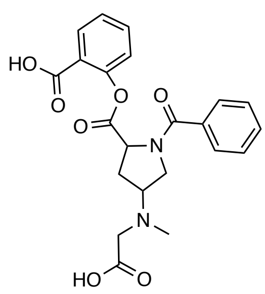

4e
0.967

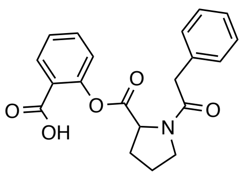

5e
1.028

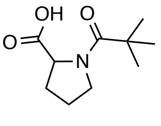

6e
1.027

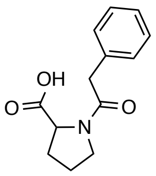

7e
1.002

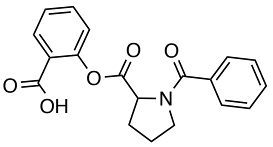

8e
0.999

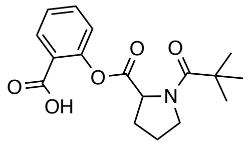

9e
0.993

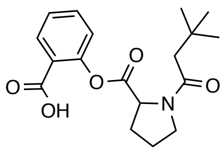

10e
0.991

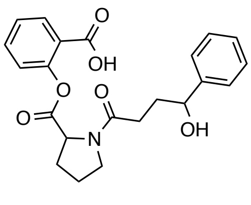

11e
0.990

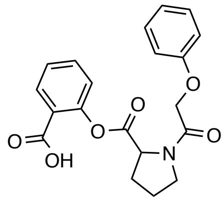

12e
0.987

Supplementary Fig. 5. Chemical structures of proline analogs and their anti-sickling activity (HbS ratio ranging from 0.967 to 1.028).

2,2 - dimethylchroman

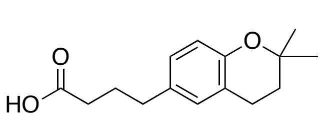

1f
1.052

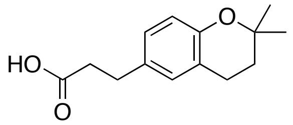

2f
1.045

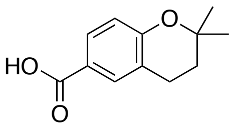

3f
1.044

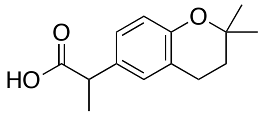

4f
1.011

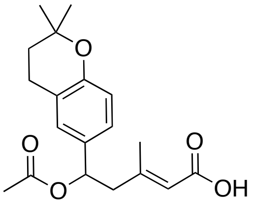

5f
1.010

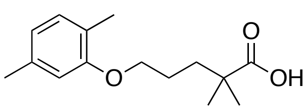

6f
1.040

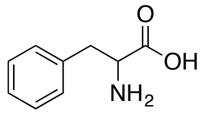

7f
1.036

Supplementary Fig. 6. Chemical structures of 2,2-dimethylchroman analogs and their anti-sickling activity (HbS ratio ranging from 1.010 to 1.052).
